# Anti‐melanogenic effects of *Medicago sativa* seed extracts on melanocytes

**DOI:** 10.1111/ics.13092

**Published:** 2025-06-17

**Authors:** Chae Rin Kim, Jung Woo, Kyu Lim Kim, Minah Choi, Hee Jung Shin, Junoh Kim, Kyung Min Lim, Chang‐Seok Lee

**Affiliations:** ^1^ Department of Senior Healthcare Eulji University Seongnam Republic of Korea; ^2^ Shinsegae International Inc Seoul Republic of Korea; ^3^ College of Pharmacy Ewha Womans University Seoul Republic of Korea; ^4^ Major in Cosmetic Science Eulji University Seongnam Republic of Korea

**Keywords:** alfalfa, artificial skin tissue, cell culture, melanogenesis, skin physiology

## Abstract

**Objective:**

Alfalfa (*Medicago sativa*) is a prominent herbal treatment among Asian countries and its antioxidant and anti‐inflammatory effects have already been generally elucidated. Excessive melanin synthesis is one of the major troubles in the cosmetics industry, thus such research has been extensively described. Here, we investigated the anti‐melanogenic effects and molecular mechanisms of two types of alfalfa extracts: alfalfa ethanol precipitate (AEP) and alfalfa ethanol supernatant (AES).

**Methods:**

The chemical composition of AEP and AES was analysed using HPAEC‐PAD and LC–MS/MS. B16F10 cells and MNT‐1 cells were used to demonstrate the inhibitory effect of two alfalfa seed extracts on melanin synthesis. The gene expression and protein levels of tyrosinase, tyrosinase‐related protein 1 (TRP1), DCT and microphthalmia‐associated factor (MITF) were confirmed using semi‐quantitative RT‐PCR, western blot and immunocytochemistry. Furthermore, the underlying mechanisms of these factors were elucidated in B16F10. The inhibitory effect on melanogenesis was validated using 3D artificial skin (MelanoDerm).

**Results:**

Both AEP and AES reduced melanin content in B16F10 cells stimulated with α‐melanocyte‐stimulating hormone (α‐MSH) and subsequently decreased mRNA and protein levels of the melanogenesis‐related targets, tyrosinase, TRP1 and MITF, as shown by semi‐quantitative RT‐PCR and immunocytochemistry. In addition, AEP and AES reduced protein levels of the MITF upstream regulators such as extracellular signal‐mitogen‐activated protein kinases (ERK), cAMP response element‐binding protein (CREB) and β‐catenin. Similar inhibition of melanin production and decreased expression of tyrosinase protein and MITF mRNA and protein were also confirmed in MNT‐1 human melanoma cells. Using artificial human skin tissue (MelanoDerm), a significant reduction in melanin content was observed.

**Conclusion:**

Alfalfa seed extracts exert an inhibitory effect on the melanin production process by modulating the activity of ERK, CREB and β‐catenin, thereby suppressing MITF and reducing the levels of tyrosinase, TRP1 and DCT. Collectively, these findings suggest that alfalfa extracts may be a promising avenue for further research and development in the fields of cosmetics and pharmaceuticals.

## Graphical abstract

Our study demonstrated that two types of alfalfa seed extract: alfalfa ethanol precipitate (AEP) and alfalfa ethanol supernatant (AES) inhibited melanogenesis through the downregulation of tyrosinase expression, following the inactivation of CREB, b‐catenin and ERK signalling pathway in α‐melanocyte‐stimulating hormone (α‐MSH)‐stimulated B16F10 mouse melanoma cells. These effects were confirmed in MNT‐1 human melanoma cells and 3D artificial skin Melanoderm, highlighting their potential as a whitening agent.

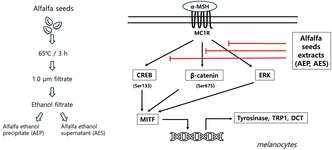

## Résumé

**Objectif:**

La luzerne (*Medicago sativa*) est une plante médicinale de premier plan originaire des pays asiatiques. Ses effets antioxydants et anti‐inflammatoires ont généralement déjà été élucidés. La synthèse excessive de mélanine est l'un des principaux problèmes au sein de l'industrie cosmétique, et cette recherche a déjà été largement décrite. Ici, nous avons étudié les effets anti‐mélanogènes et les mécanismes moléculaires de deux types d'extraits de luzerne: le précipité d'éthanol de luzerne (*alfalfa ethanol precipitate*, AEP) et le surnageant d'éthanol de luzerne (*alfalfa ethanol supernatant*, AES).

**Méthodes:**

La composition chimique de l'AEP et de l'AES a été analysée par HPAEC‐PAD et LC–MS/MS. Les cellules B16F10 et les cellules MNT‐1 ont été utilisées afin de démontrer l'effet inhibiteur de deux extraits de graines de luzerne sur la synthèse de la mélanine. L'expression génétique et les niveaux de protéines de la tyrosinase, de la protéine 1 liée à la tyrosinase (*tyrosinase‐related protein 1*, TRP1), de la DCT et du facteur associé à la microphtalmie (*microphthalmia‐associated factor*, MITF) ont été confirmés à l'aide de tests RT‐PCR semi‐quantitatifs, tests Western Blot et d'immunocytochimie. En outre, les mécanismes sous‐jacents de ces facteurs ont été élucidés dans les cellules B16F10. L'effet inhibiteur sur la mélanogénèse a été validé à l'aide d'une peau artificielle en 3D (MelanoDerm).

**Résultats:**

L'AEP et l'AES ont tous deux réduit la teneur en mélanine des cellules B16F10 stimulées par l'hormone stimulant l'α‐mélanocytaire (*α‐melanocyte‐stimulating hormone*, α‐MSH) et ont ensuite diminué les niveaux d'ARNm et de protéines des cibles liées à la mélanogénèse, la tyrosinase, le TRP1 et le MITF, comme l'ont montré les tests RT‐PCR semi‐quantitatifs et de l'immunocytochimie. En outre, l'AEP et l'AES ont réduit les niveaux de protéines des régulateurs en amont du MITF, tels que les protéines kinases activées par le signal mitogène extracellulaire (ERK), la protéine de liaison de l'élément de réponse cAMP (*cAMP response element‐binding protein*, CREB) et la β‐caténine. Une inhibition similaire de la production de mélanine ainsi qu'une diminution de l'expression de la protéine tyrosinase et de l'ARNm et de la protéine du MITF ont également été confirmées dans la cellule de mélanome humain MNT‐1. Une réduction significative de la teneur en mélanine a été observée sur du tissu de peau humaine artificielle (MelanoDerm).

**Conclusion:**

Les extraits de graines de luzerne exercent un effet inhibiteur sur le processus de production de mélanine en modulant l'activité d'ERK, de CREB et de β‐caténine, supprimant ainsi le MITF et réduisant les niveaux de tyrosinase, de TRP1 et de DCT. Collectivement, ces résultats suggèrent que les extraits de luzerne peuvent être une voie prometteuse pour la recherche et le développement dans les domaines des cosmétiques et des produits pharmaceutiques.

## INTRODUCTION

Melanogenesis, a unique characteristic of melanocytes, is a self‐defence mechanism against extrinsic threats such as ultraviolet radiation. Melanin, in the form of melanosome, is delivered—via dendrites to keratinocytes where its pigment is revealed. Tyrosine, the common melanin precursor, is transformed into L‐dopa quinone by tyrosinase, and then converted through a series of steps to eumelanin (brown–black) or pheomelanin (yellow‐red) [1, 2]. Through typically an efficient process, excessive melanin production can cause abnormal skin disorders, including melasma, melanoma, freckles and lentigo [3].

Tyrosinase plays a pivotal role in melanogenesis; hence, inhibiting its enzyme activity or reducing its gene or protein level could be a promising strategy for enhancing whitening efficacy. Microphthalmia‐associated transcription factor (MITF), a bHLH‐Zip (basic helix–loop–helix leucine zipper) transcription factor, which is a master regulator of genes encoding tyrosinase and tyrosinase‐related protein‐1 (TRP1 or TYRP1) and ‐2 (TYRP2, also known as dopachrome tautomerase [DCT]), is a major regulator of melanocyte differentiation and development. The MITF gene is regulated by numerous transcription factors, including cAMP response element‐binding protein (CREB), β‐catenin and SOX10 [4, 5, 6]. Upon phosphorylation of its Ser133 residue, CREB becomes activated and organizes a CBP/CREB complex at the MITF promoter [7]. Phosphorylation of Ser133 can be regulated by diverse pathways. Notably, α‐melanocyte‐stimulating hormone (α‐MSH) binds to the membrane protein, melanocortin 1 receptor (MC1R), thereby activating downstream cAMP‐protein kinase A (PKA) signalling [8]. Preliminary observations indicate that, in the presence of ultraviolet B (UVB) illumination, the c‐KIT‐p38MAPK (mitogen‐activated protein kinase) pathway can also regulate the transcriptional activity of CREB [9, 10]. ERK (extracellular‐regulated kinase), another member of the MAPK family, promotes the transcriptional activity of CREB through its downstream signalling factors, mitogen‐ and stress‐activated protein kinase 1 (MSK‐1) or p90 ribosomal S6 kinase (p90RSK) [11]. CREB activity can also be reduced by attenuating the PI3K‐AKT signalling, which acts through glycogen synthase kinase‐3 β (GSK3β) to phosphorylate CREB at a different site and inhibit it [12]. β‐catenin binds to the Lef‐1 binding site in the MITF promoter and initiates downstream gene expression [13].

According to prior studies, the activity of MITF can be altered by its phosphorylation status. For instance, dual phosphorylation of Ser73 and Ser409 negatively regulates MITF levels by promoting proteasome‐mediated degradation. However, when MITF is phosphorylated only at Ser73, it promotes the interaction with MITF co‐activator p300‐CBP and enhances the transcriptional activation [14, 15, 16].

Alfalfa (*Medicago sativa L*.), a member of the Fabaceae family, has been widely used as an herbal treatment throughout Asian countries [17, 18]. Previous investigations have shown that alfalfa extracts have a high content of bioactive compounds such as flavonoids, alkaloids, saponins and phenolic compounds [17, 19], and have demonstrated the antioxidant and anti‐inflammatory efficacy of alfalfa seeds, leaves and flower extracts [20, 21, 22]. However, whether alfalfa extracts possess anti‐melanogenic effects has not been investigated. Here, we explored the anti‐melanogenic effects of two types of alfalfa seed extract: alfalfa ethanol precipitate (AEP) and alfalfa ethanol supernatant (AES).

## MATERIALS AND METHODS

### Preparation of AEP and AES

In brief, alfalfa seeds were ground and extracted with purified water at 65°C for 3 h. Impurities were removed after extraction by passing the mixture through a 1.0‐μm filter. The filtered extract was slowly added to ethanol with stirring. The precipitate (AEP) was collected, washed with ethanol, dried and ground using a mortar and pestle. The filtrate was concentrated at 60°C using a rotary evaporator (Eyela, Bohemia, NY, USA). The concentrated extract (AES) was homogenized with an ethanol‐sterilized mortar and pestle. Both AEP and AES subsequently used for experiments were kindly supplied from Durae (Anyang‐si, Gyeonggi‐do, Republic of Korea).

### Cell culture

B16F10 cells, purchased from American Type Culture Collection (ATCC, Manassas, VA, USA), were cultured in Dulbecco's Modified Eagle Medium (DMEM; Welgene, Gyeongsan‐si, Gyeongsangbuk‐do, Republic of Korea) supplemented with 5% fetal bovine serum (FBS; 30–2020; ATCC) and 1% penicillin–streptomycin mixture. MNT‐1 cells, kindly provided by Prof. Kyungmin Lim (Ewha Women's University, Seoul, Republic of Korea), were cultured in Modified Essential Medium (MEM; Hyclone, Logan, UT, USA) containing 10% DMEM (Hyclone), 20% FBS (Hyclone), 1% penicillin–streptomycin mixture (Hyclone) and 1% HEPES (Gibco, Grand Island, NY, USA). All cells were incubated at 37°C in a humidified 5% CO_2_ environment and cultured until passage 5~20 (3~4 d per passage).

### Cell viability assay

Cell viability was determined using a Quanti‐MAX WST‐8 Cell Viability Assay Kit (Biomax, Guri‐si, Gyeonggi‐do, Republic of Korea) according to the manufacturer's protocol. Cells were seeded in 96‐well plates (SPL, Pocheon‐si, Gyeonggi‐do, Republic of Korea) and stabilized for 24 h, then treated with AEP or AES in a various concentrations and incubated for 72 h. Medium was then removed and replaced with medium containing 10% WST‐8 solution. After incubation for an additional 2 h, absorbance at 450 nm was measured.

### Measurement of melanin content

The protocol for assessing inhibition of melanin production was adapted from pioneering research, with modification [23, 24]. Briefly, B16F10 cells were seeded on a 48‐well plate (SPL) at a density of 2.5 × 10^4^ cells per well. After 24 h, cells were treated with AEP or AES in the presence of α‐MSH (200 nM) (Sigma, St. Louis, MI, USA) and incubated for 72 h. Aliquots (100 μL) of supernatants were collected and extracellular melanin content was determined by measuring absorbance at 405 nm using a microplate reader (BioTek, Winooski, VT, USA). For measurement of intracellular melanin content, medium was removed, and cells were dissolved by adding 1 N NaOH to each well and heating at 60°C for 30 min. Lysate was transferred to a 96‐well plate, and absorbance was measured at 405 nm as described above. MNT‐1 cells were seeded on 24‐well plates at a density of 8 × 10^4^ cells per well and treated with AEP or AES for 96 h. Subsequent procedures were the same as those described for B16F10 cells.

### Real‐time PCR

For extraction of RNA, B16F10 and MNT‐1 cells were seeded in 6‐well plates and incubated with AEP or AES, with or without α‐MSH (200 nM) for each time zone. Total RNA was extracted with TRIzol reagent (Thermo‐Fisher Scientific, Cleveland, OH, USA) according to the manufacturer's instructions. cDNA was synthesized from total mRNA using the GoScript Reverse Transcription System (A5003; Promega, Madison, WI, USA) and a thermal cycler (BioRad, Hercules, CA, USA). Tyrosinase (qMmuCED0045245, qHsaCED0042402, BioRad), TRP1 (qMmuCID0015969, qHsaCID0021072, BioRad) and MITF (qMmuCID0013258, qHsaCED0037870, BioRad) were amplified from cDNA by real‐time quantitative polymerase chain reaction (qRT‐PCR), and their relative expression levels were determined using the CFX Connect Optics Module (BioRad). Conditions of qRT‐PCR were performed for a total of 40 cycles. PCR conditions were as follows: denaturation at 95°C for 10 s, annealing at 60°C for 10 s and extension at 72°C for 30 s. All results were normalized to β‐actin mRNA levels (BioRad).

### Western blot analysis

Cells were washed twice with phosphate‐buffered saline (PBS) and lysed with RIPA buffer (9806S; Cell Signaling Technology, Danvers, MA, USA) containing phosphatase and protease inhibitors. Lysates were then centrifuged at 13 000 rpm at 4°C for 20 min and supernatants were collected. The protein content was quantified by BCA protein assay kit (Thermo‐Fisher Scientific). Quantified proteins were separated by SDS‐PAGE and transferred to a nitrocellulose membrane (BioRad). The membrane was blocked by incubating for at least 2 h in PBS containing 5% Blotting‐Grade Blocker (BioRad). Each membrane was incubated at 4°C with primary antibodies against tyrosinase (ab180753; Abcam, Cambridge, UK), TRP1 (sc‐25 543; Santa Cruz Biotechnology, Dalla, TX, USA), MITF (13092‐1‐AP; Proteintech, Chicago, IL, USA), phosphorylated ERK (p‐ERK; 9101S; Cell Signaling Technology), total ERK (t‐ERK; 9102S; Cell Signaling Technology), p‐CREB (9198S; Cell Signaling Technology), t‐CREB (9197S; Cell Signaling Technology), p‐β‐catenin (Ser675) (9567S; Cell Signaling Technology), t‐β‐catenin (ab16051; Abcam) and β‐actin (ab179467; Abcam). On the following day, the membrane was washed with Tris‐buffered saline containing 0.1% Tween 20 (TBST) and incubated with goat anti‐rabbit IgG H&L secondary antibodies (ab6721; Abcam). Immunoreactive proteins were visualized using ECL substrate (BioRad).

### Immunocytochemistry

B16F10 cells seeded on cell culture slides (SPL) and MNT‐1 cells seeded on 18 × 18 mm coverslips were treated with AEP or AES, with or without α‐MSH (200 nM), as described above. Cells were fixed with 4% paraformaldehyde (Bylabs, Hansam‐si, Gyeonggi‐do, Republic of Korea) for 30 min at 4°C and then permeabilized with 0.1% Triton X‐100 (Sigma) dissolved in PBS. Cells were washed twice in PBS, blocked with 3% bovine serum albumin (BSA), and incubated with primary antibodies against tyrosinase (MA5‐14177; Thermo‐Fisher Scientific) and MITF (13092‐1‐AP; Proteintech) overnight at 4°C. The following day, immunoreactive proteins in samples were detected by incubating with fluorescence‐conjugated secondary antibodies (4412S, 8890S; Cell Signaling Technology), and nuclei were counterstained with 4’,6‐diamidino‐2‐phenylindole (DAPI; ab104139; Abcam). Results were visualized by fluorescence microscopy.

### Evaluation of skin‐whitening efficacy using a 3D reconstructed skin model

MelanoDerm (MatTek Corp., Ashland, MA, USA), a three‐dimensional skin model composed of normal human keratinocytes and melanocytes that mimic the structure and function of the human epidermis, was used as a more physiological test system. For these tests, MelanoDerm was pre‐cultured for 24 h and then incubated with each compound for 6 d. A lightness/darkness index (Δ*L*) value was determined using Adobe Photoshop CC 2015 software (San Jose, CA, USA). For histological examinations, tissues were fixed by incubating with phosphate‐buffered formalin (PFA) for 24 h and then stained with haematoxylin and eosin (H&E).

### Statistical analysis

The data are presented as means ± standard deviation (SD), and the significance of differences between the three independent values was determined using Student's *t*‐test.

## RESULTS

### Determination of monosaccharide and chemical composition of AEP and AES

To determine the monosaccharide composition of the polysaccharides obtained from alfalfa seed extracts, we performed high‐performance anion‐exchange chromatography with pulsed amperometric detection (HPAEC‐PAD) after the acidic hydrolysis procedure. For both extracts, the highest amount was found for galactose, which made up 0.425 mg/mg and 0.084 mg/mg of monosaccharides in AEP and AES, respectively. This was followed by mannose (0.304 mg/mg), glucose (0.002 mg/mg) and arabinose (0.001 mg/mg) in AEP, and glucose (0.024 mg/mg), rhamnose (0.004 mg/mg), arabinose (0.002 mg/mg) and mannose (0.001 mg/mg) in AES (Tables S1–S3).

γ‐Aminobutyric acid (GABA), an inhibitory neurotransmitter that acts on the central nervous system of mammals, plays a crucial role in reducing neuronal excitability and maintaining neural network stability [25, 26]. Molagoda et al. showed that GABA exerts whitening efficacy through the cAMP‐CREB pathway [27]. Recent studies have suggested that protocatechuic acid (PCA), a naturally synthesized phenolic acid enriched in various fruits and vegetables, possesses well‐established pharmacological activities including antioxidant, anticancer and antidiabetic activity [28, 29]. Recent works have suggested that PCA inhibits melanin synthesis [30, 31]. Ferulic acid is a well‐known type of phenolic acid, with widely demonstrated antioxidant efficacy, and has recently been researched on whitening efficacy [32, 33]. To detect and quantify these three indicator compounds, we performed liquid chromatography with tandem mass spectrometry (LC–MS/MS). This analysis revealed that of these compounds, GABA had the highest concentration in AEP (131.57 mg/kg), followed by PCA (13.57 mg/kg) and ferulic acid (0.72 mg/kg). Although the rank order of these compounds in AES was the same, the concentrations were much higher: GABA 4576.66 mg/kg, PCA 532.47 mg/kg and ferulic acid 28.39 mg/kg (Table SIV, SV). Detailed procedures of HPAEC‐PAD and LC–MS/MS are described in supplementary materials.

### AEP and AES inhibit melanin production in α‐MSH‐stimulated B16F10 cells

**FIGURE 1:**
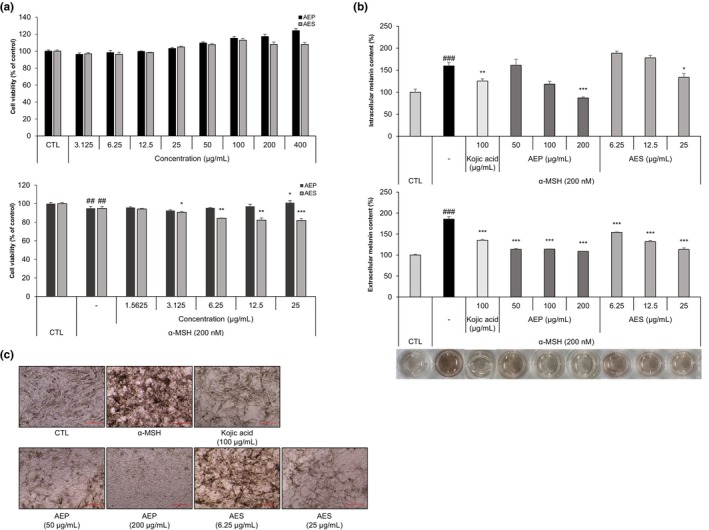
Alfalfa ethanol precipitate (AEP) and alfalfa ethanol supernatant (AES) attenuate melanin production in α‐ melanocyte‐stimulating hormone (MSH)‐stimulated B16F10 cells. (a) Cytotoxicity of AEP and AES determined using WST‐8 assays. B16F10 cells were treated with AEP or AES alone at concentrations of 3.125–400 μg/mL, or at 1.5625–25 μg/mL together with 200 nM α‐MSH for 72 h. (b) B16F10 cells were treated with kojic acid (100 μg/mL), AEP (50–200 μg/mL) or AES (6.25–25 μg/mL), with or without α‐MSH (200 nM) for 72 h in phenol red‐free medium. Melanin content was determined by measuring absorbance at 405 nm. (c) Morphology of α‐MSH‐stimulated B16F10 cells treated with AEP (50, 200 μg/mL) or AES (6.25, 25 μg/mL), assessed using optical microscopy. Scale bar = 100 μm. Data are presented as means ± SD (^##^ *p* < 0.001, ^###^ *p* < 0.001 vs. control group; *p < 0.05, **p < 0.01, ***p < 0.001 vs. α‐MSH treatment group).

Before assessing the effects of alfalfa extracts on melanin production, we determined their cytotoxicity by performing WST‐8 assays after treating B16F10 cells with different concentrations of AEP or AES for 3 d in the presence or absence of α‐MSH. Neither AEP nor AES, when applied alone from 3.125 to 400 μg/mL, showed any significant effects on cells. With α‐MSH (200 nM) co‐treatment, AES showed slight toxicity at 25 μg/mL, whereas AEP did not (Figure 1a). Then we evaluated the efficacy of AEP and AES in inhibiting melanin production and found that both reduced intracellular melanin content in a dose‐dependent manner. Notably, at a concentration of 200 μg/mL, AEP inhibited the production of melanin to a greater extent than the positive control, kojic acid (100 μg/mL). We also evaluated extracellular melanin content by measuring the absorbance of cell supernatants, and similarly found that both AEP and AES brightened the α‐MSH‐treated medium (Figure 1b,c).

### AEP and AES suppress melanogenic gene expression and protein level in α‐MSH‐stimulated B16F10 cells

**FIGURE 2:**
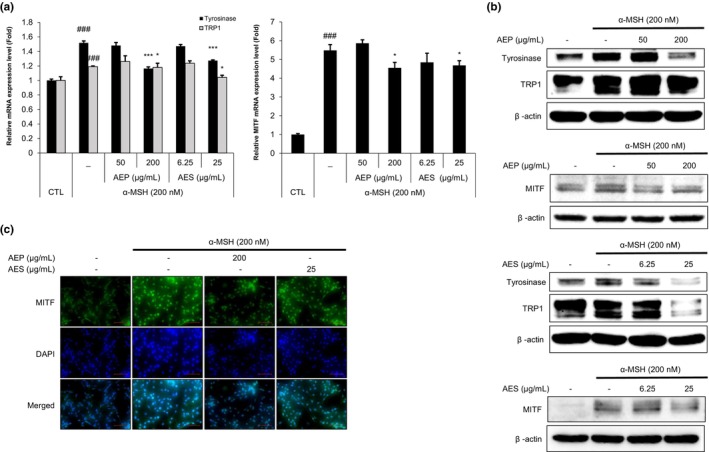
Alfalfa ethanol precipitate (AEP) and alfalfa ethanol supernatant (AES) attenuate the transcription and protein level of melanogenesis‐related protein of α‐ melanocyte‐stimulating hormone (MSH)‐stimulated B16F10 cells. (a) mRNA levels of tyrosinase, tyrosinase, tyrosinase‐related protein 1 (TRP1) and microphthalmia‐associated factor (MITF). Tyrosinase and TRP1 mRNA levels were analysed after treatment with AEP (50, 200 μg/mL) or AES (6.25, 25 μg/mL) for 24 h. MITF was analysed after treatment for 2 h. (b) Protein levels of tyrosinase, TRP1 and MITF, determined by semi‐quantitative Western blotting. (c) Immunocytochemistry of MITF in α‐MSH‐stimulated B16F10 cells treated with AEP (200 μg/mL) or AES (25 μg/mL). Scale bar = 50 μm. Data are presented as means ± SD (^###^ *p* < 0.001 vs. control group; *p < 0.05, ***p < 0.001 vs. α‐MSH treatment group).

To investigate whether AEP and AES affect the expression of melanogenesis‐related genes, we performed qRT‐PCR. Tyrosinase and TRP1, which play pivotal roles in melanin synthesis, were upregulated by treatment with α‐MSH (200 nM). Both genes were attenuated by AES, whereas AEP reduced only tyrosinase levels (Figure 2a). Moreover, expression levels of the gene encoding MITF, a crucial transcriptional regulator of tyrosinase and TRP1, were downregulated by AEP and AES (Figure 2a). In line with qRT‐PCR data, tyrosinase, TRP1 and MITF protein levels were clearly decreased in α‐MSH‐stimulated B16F10 cells (Figure 2b; Figure S1a,b). Both Western blot and immunofluorescence analyses showed that MITF levels in α‐MSH‐stimulated B16F10 cells were reduced by both AEP (200 μg/mL) and AES (25 μg/mL) compared with α‐MSH (200 nM) treatment alone (Figure 2c). This significant attenuation of melanogenesis‐related factors indicates that AEP and AES display anti‐melanogenic effects.

### AEP and AES regulate melanogenesis via ERK, β‐catenin and CREB pathways

**FIGURE 3:**
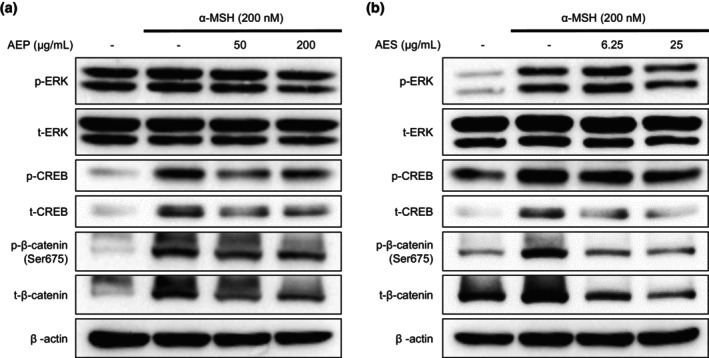
Effect of alfalfa ethanol precipitate (AEP) and alfalfa ethanol supernatant (AES) on extracellular‐regulated kinase (ERK), cAMP response element‐binding protein (CREB) and β‐catenin. (a), (b) α‐ melanocyte‐stimulating hormone (MSH)‐stimulated B16F10 cells were treated with AEP (50, 200 μg/mL) or AES (6.25, 25 μg/mL) for 30 min and analysed by Western blotting.

We next explored the molecular mechanisms underlying the actions of AEP and AES. Expression levels of the MITF gene depend on the transcription factors, CREB and β‐catenin [5, 13]. β‐catenin exists in two forms, inactivated and activated, with translocation into the nucleus depending on its phosphorylation status. Specifically, phosphorylation of β‐catenin at Ser675 is a well‐known signal for β‐catenin nuclear translocation and enhanced transcriptional activity [34, 35]. Both AEP and AES reduced total β‐catenin expression as well as levels of Ser675‐phosphorylated β‐catenin (Figure 3a,b; Figure S2a,b). Notably, they also decreased the phosphorylation of ERK, which increases the transcriptional activity of MITF by phosphorylating Ser73 and recruiting the transcriptional cofactor, p300‐CBP [36]. AEP slightly downregulated phosphorylation of CREB at 50 μg/mL, whereas AES decreased CREB phosphorylation in a concentration‐dependent manner (Figure 3a,b; Figure S2a,b). Collectively, these findings indicate that both alfalfa extracts modulate molecular mechanisms that regulate the expression level and activity of MITF.

### AEP and AES repress the melanin contents of MNT‐1 cells and artificial human skin

**FIGURE 4:**
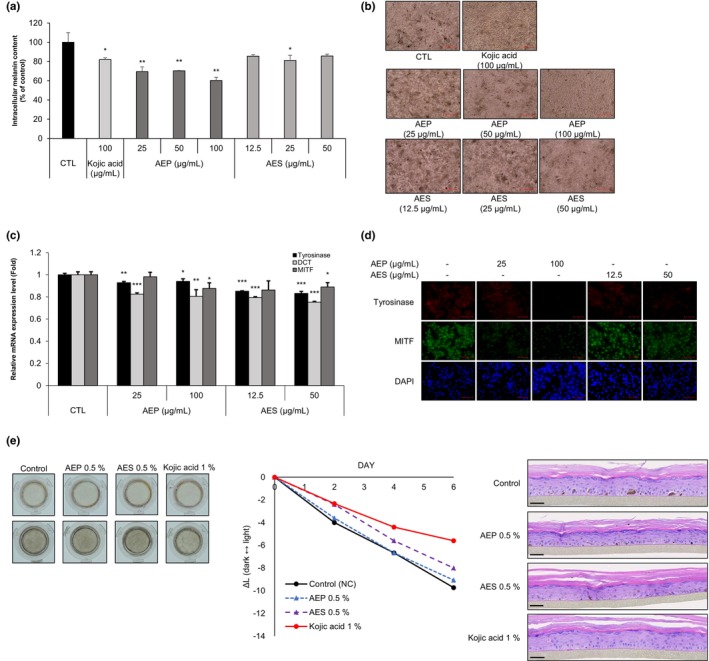
Inhibitory effect of alfalfa ethanol precipitate (AEP) and alfalfa ethanol supernatant (AES) on human MNT‐1 cells and the MelanoDerm artificial skin model. (a) MNT‐1 cells were treated with AEP (25–100 μg/mL), AES (12.5–50 μg/mL) or kojic acid (100 μg/mL) for 96 h. Melanin content was determined by measuring absorbance at 405 nm. (b) Morphological changes in MNT‐1 cells treated with AEP (25–100 μg/mL), AES (12.5–50 μg/mL) or kojic acid (100 μg/mL). Scale bar = 100 μm. (c) Inhibitory effect of AEP and AES on mRNA levels of tyrosinase, DCT and microphthalmia‐associated factor (MITF) in MNT‐1 cells. Cells were treated with AEP (25–100 μg/mL) or AES (12.5–50 μg/mL) for 48 h. (d) Inhibitory effect of AEP (25–100 μg/mL) and AES (12.5–50 μg/mL) on tyrosinase and MITF protein expression in MNT‐1 cells, determined by immunocytochemistry. Scale bar = 50 μm. (e) Skin‐whitening effects of AEP (0.5%) and AES (0.5%) on the artificial human skin tissue model, MelanoDerm. Scale bar = 50 μm. Data are presented as means ± SD (*p < 0.05, **p < 0.01, ***p < 0.001 vs. control group).

To clarify the anti‐melanogenic effect of AEP and AES in a human experimental system, we assayed melanin content in highly pigmented MNT‐1 human melanoma cells. Both AEP and AES significantly inhibited melanin synthesis compared with the control group (Figure 4a). Microscopy analyses further showed that AEP (100 μg/mL) and AES (50 μg/mL) exhibited whitening effects similar to the positive control, kojic acid (100 μg/mL) (Figure 4b). Furthermore, both AEP and AES decreased mRNA levels of tyrosinase, DCT and MITF (Figure 4c). Immunofluorescence analysis confirmed the decrease in tyrosinase and MITF expression (Figure 4d).

To further validate these anti‐melanogenic effects, we used MelanoDerm, a highly pigmented, artificial human skin model that is widely applied to evaluate alterations in pigment levels. The brightness of the skin tissue was measured and converted into Δ*L* values using image analysis; tissue was also stained with H&E for histological analysis. After 6 d of treatment, untreated tissue appeared darker compared with that at day 0. In contrast, the tissue colour was lighter following treatment with AEP or AES (each at 0.5%), and Δ*L* value was reduced compared with the untreated group (Figure 4e).

## DISCUSSION

The biological pigment, melanin, is a unique membrane‐bound compound located within specialized organelles called melanosomes. Melanin serves both mechanical and biological functions, protecting cells from degradation, oxidative stress and apoptosis caused by sunlight irradiation [37, 38, 39]. UV radiation is a ubiquitous environmental factor, essential for living things. Yet, immoderate irradiation can cause melasma, brown spots and freckles [40]. More seriously, it can also be a cause of skin tumours, such as malignant melanoma [39, 41]. Recent reports have highlighted possible side effects of some whitening ingredients, raising interest in developing novel anti‐melanogenic candidates from natural sources [42, 43]. Alfalfa is an extensively cultivated crop among livestock farms owing to its high nutritional value, high yield and adaptability to poor environmental conditions [44, 45]. In the current study, two types of alfalfa seed extract exerted significant melanin inhibition in mouse melanoma cells in association with decreased protein and mRNA levels of the melanogenesis‐related factors, tyrosinase, TRP1 and MITF.

In the presence of UV light, keratinocytes and melanocytes synthesize α‐MSH, which upregulates the MC1R‐cAMP‐related pathway [46, 47]. CREB, downstream factor of MC1R, is activated upon phosphorylation at Ser133, enhancing transcriptional activity such as MITF [8]. As shown in Figure 3, AEP and AES downregulated the protein level of p‐CREB. As prior studies have described, MITF's transcription activity also can be regulated by β‐catenin [48]. The expression and stability of β‐catenin can be regulated by upstream factor GSK‐3β. Among the various phosphorylation residues of β‐catenin, phosphorylation at Ser33, Ser37 and Thr41 by GSK‐3β is recognized as an initiation signal for degradation [49, 50]. On the contrary, phosphorylation at Ser675 and Ser552 is noticed to be the signal for nuclear translocation.

In this study, we found that AEP and AES suppressed protein levels of both total β‐catenin and Ser675‐phosphorylated β‐catenin. According to prior studies, activated GSK‐3β lessens phosphorylation of CREB [12, 51]. Our results indicate that CREB phosphorylation was slightly decreased by AEP at 50 μg/mL, whereas AES downregulated CREB phosphorylation in a concentration‐dependent manner. Taken together, our results suggest that the efficacy of AEP and AES in suppressing protein levels of p‐CREB and β‐catenin involves a GSK‐3β‐related mechanism, but further verification is required.

The MAPK family members, p38, JNK and ERK, are known to control the stability and transcriptional activity of MITF through disparate and related mechanisms [52, 53, 54]. ERK‐mediated phosphorylation of MITF enhances the transcriptional activity or proteasome‐mediated degradation of MITF, depending on which residue is phosphorylated and the specific downstream factors involved [36, 55, 56]. Because MITF is the most notable transcription factor involved in the melanin production process, controlling its activity and expression level is a major strategy for developing whitening agents. Our experiments show that both AEP and AES reduced levels of phosphorylated (activated) ERK, suggesting that the ERK pathway is involved in the downregulation of MITF.

Sugars are well‐known natural compounds found ubiquitously in the natural environment, and previous studies have reported that sugar and sugar‐related compounds are promising anti‐melanogenic agents [57, 58]. One of the most abundant sugars in nature is galactose, which is a component of several plant cell wall polymers. The galactose derivative, 3,6‐anhydro‐l‐galactose (L‐AHG) has been reported to have whitening effects [59, 60, 61]. Based on the pioneering experiments, our results showed that AEP and AES exhibit similar anti‐melanogenic effects, which were considered to be attributable to the high concentrations of galactose that both share. On the other hand, previous studies have reported that GABA exhibits whitening effects through regulation of the cAMP‐CREB pathway [27]. In this paper, LC–MS/MS analyses indicated that the GABA content of AES was about ~30‐fold higher than that of AEP and further showed that this increased GABA content can be associated with a greater whitening effect of AES, even at a lower concentration than AEP. Taken together, these observations suggest that the whitening efficacy of AES through downregulation of the CREB pathway could reflect its high concentration of GABA. Nonetheless, further investigations are required to elucidate the active components of AEP and AES required for their anti‐melanogenic efficiency.

Overall, our results show that both AEP and AES exert significant anti‐melanogenic effects through regulation of melanogenic enzymes and their upstream factors, CREB, β‐catenin and ERK. Accordingly, we suggest that alfalfa seed extracts could be promising raw materials for natural melanogenic candidates.

## CONFLICT OF INTEREST STATEMENT

The authors declare no conflict of interest.

## Supporting information


**Table S1.** Monosaccharide analysis of standard.
**TABLE S2**. Monosaccharide analysis of alfalfa ethanol precipitate (AEP).
**TABLE S3**. Monosaccharide analysis of alfalfa ethanol supernatant (AES).
**TABLE S4**. Results of indicator analysis (γ‐aminobutyric acid (GABA), protocatechuic acid and ferulic acid) of AEP.
**TABLE S5**. Results of indicator analysis (γ‐aminobutyric acid (GABA), protocatechuic acid and ferulic acid) of AES.
**FIGURE S1**. Alfalfa ethanol precipitate (AEP) and alfalfa ethanol supernatant (AES) regulate the protein level of melanogenic proteins in α‐melanocyte‐stimulating hormone (MSH)‐stimulated B16F10 cells. (a), (b) Densitometry of tyrosinase, tyrosinase‐related protein 1 (TRP1) and microphthalmia‐associated factor (MITF) in AEP (50, 200 μg/mL), AES (6.25, 25 μg/mL) treated α‐MSH‐stimulated B16F10 cells. (^#^ *p* < 0.05, ^###^ *p* < 0.001, vs. control group, ***p < 0.001 vs. α‐MSH treatment group).
**FIGURE S2**. Determination of molecular mechanisms of alfalfa ethanol precipitate (AEP) and alfalfa ethanol supernatant (AES). (a), (b) Densitometry of p‐extracellular‐regulated kinase (ERK), p‐ cAMP response element‐binding protein (CREB), t‐β‐catenin and p‐β‐catenin (Ser675) in AEP (50, 200 μg/mL), AES (6.25, 25 μg/mL) treated α‐ melanocyte‐stimulating hormone (MSH)‐stimulated B16F10 cells. (c) The cytotoxicity of AEP and AES on MNT‐1 cells were determined by using WST‐8 assay. Cells were treated with AEP or AES in a concentration range of 3.125–200 μg/mL for 96 h. ((a), (b): ^##^ *p* < 0.01, ^###^ *p* < 0.001, vs. control group, *p < 0.05, **p < 0.01, ***p < 0.001 vs. α‐MSH treatment group, (c): **p < 0.01 vs. control group).
