# Supplementary material for: Anti‐melanogenic effects of Medicago sativa seed extracts on melanocytes
Source: Int J Cosmet Sci. 2025 Jun 17;47(6):1005–16. doi: 10.1111/ics.13092 (PMC12666729; doi:10.1111/ics.13092)
Supplement: Supplementary file 1 — Table S1. Monosaccharide analysis of standard. TABLE S2. Monosaccharide analysis of alfalfa ethanol precipitate (AEP). TABLE S3. Monosaccharide analysis of alfalfa ethanol supernatant (AES). TABLE S4. Results of indicator analysis (γ‐aminobutyric acid (GABA), protocatechuic acid and ferulic acid) of AEP. TABLE S5. Results of indicator analysis (γ‐aminobutyric acid (GABA), protocatechuic acid and ferulic acid) of AES. FIGURE S1. Alfalfa ethanol precipitate (AEP) and alfalfa ethanol supernatant (AES) regulate the protein level of melanogenic proteins in α‐melanocyte‐stimulating hormone (MSH)‐stimulated B16F10 cells. (a), (b) Densitometry of tyrosinase, tyrosinase‐related protein 1 (TRP1) and microphthalmia‐associated factor (MITF) in AEP (50, 200 μg/mL), AES (6.25, 25 μg/mL) treated α‐MSH‐stimulated B16F10 cells. (# p < 0.05, ### p < 0.001, vs. control group, ***p < 0.001 vs. α‐MSH treatment group). FIGURE S2. Determination of molecular mechanisms of alfalfa ethanol precipitate (AEP) and alfalfa ethanol supernatant (AES). (a), (b) Densitometry of p‐extracellular‐regulated kinase (ERK), p‐ cAMP response element‐binding protein (CREB), t‐β‐catenin and p‐β‐catenin (Ser675) in AEP (50, 200 μg/mL), AES (6.25, 25 μg/mL) treated α‐ melanocyte‐stimulating hormone (MSH)‐stimulated B16F10 cells. (c) The cytotoxicity of AEP and AES on MNT‐1 cells were determined by using WST‐8 assay. Cells were treated with AEP or AES in a concentration range of 3.125–200 μg/mL for 96 h. ((a), (b): ## p < 0.01, ### p < 0.001, vs. control group, *p < 0.05, **p < 0.01, ***p < 0.001 vs. α‐MSH treatment group, (c): **p < 0.01 vs. control group). [file ICS-47-1005-s001.docx]

**Supplementary materials**

**Materials and Methods**

**Determination of Monosaccharide composition in the AEP and AES**

Monosaccharide composition was analyzed using a high-performance anion-exchange chromatography with pulsed amperometric detection (HPAEC-PAD) system (Dionex, Sunnyvale, CA, USA) using a gold working electrode and an Ag/AgCl reference electrode. Separation was achieved using a CarboPac ™ PA1 analytical column (Thermo-Fisher Scientific, Cleveland, OH, USA), and the column temperature was set at 35℃. The mobile phase (eluent) were 18 mM NaOH and 200 mM NaOH and the analyses were performed at a 1.0 mL/min flow rate. The column was equilibrated with 18 mM NaOH before sample injection. A 20 μL sample was injected into the system for each analysis. Standard curves were generated using monosaccharides including fucose, rhamnose, arabinose, galactose, glucose, xylose, fructose, and mannose (Sigma, St. Louis, MI, USA).

**Determination of γ-aminobutyric acid (GABA)**

The quantification of γ-aminobutyric acid (GABA) was performed using liquid chromatography-tandem mass spectrometry (LC-MS/MS) with a Qtrap 4500 system (SCIEX, Framingham, MA, USA). For the standard curve, GABA (Sigma Aldrich) was diluted in 50 % methanol and prepared in various concentrations. For sample preparation, each sample was extracted with 50% methanol with 30 minutes of ultrasonication followed by dilution with 50% methanol to a final concentration corresponding to a 2,000-fold dilution for AEP and a 100,000-fold dilution for AES. Chromatographic separation was achieved using a Luna Omega polar C18 (2.1 mm × 150 mm, 3 μm; Phenomenex, Torrance, CA, USA). The mobile phase consisted of 0.1% formic acid in distilled water. The flow rate was 0.25 mL/min, and the column temperature was maintained at 25°C. Electrospray ionization (ESI) in positive ion mode was subsequently applied for the analysis. The monitored transitions were *m/z* 104.02 → 87.00 and 69.10.

**Determination of protocatechuic acid and ferulic acid**

Quantitation of protocatechuic acid and ferulic acid was performed by LC-MS/MS using a Qtrap 4500 system (SCIEX). Samples were injected into an Agilent Poroshell EC C18 column (2.1 mm × 150 mm, 2.7 μm; Agilent Technologies, Santa Clara, CA, USA) with a mobile phase consisting of 0.1% acetic acid in distilled water (solvent A) and 0.1% acetic acid in acetonitrile (solvent B). The temperature was set at 35℃, and the flow rate was 0.25 mL/min for the protocatechuic acid and 0.3 mL/min for the ferulic acid. After the chromatographic separation, ESI in negative ion mode was applied. Multiple reaction monitoring (MRM) transitions were monitored as follows: *m/z* 192.97 → 133.90, 177.90, 149.00 for ferulic acid, and *m/z* 152.87 → 108.90, 108.00, 91.00 for protocatechuic acid. For the standard curve, protocatechuic acid and ferulic acid (both from Sigma-Aldrich) were diluted with 50% methanol and prepared at various concentrations, following the same procedure as for GABA. Each sample was extracted with 50% methanol using ultrasonication for 30 minutes and diluted with methanol. For protocatechuic acid analysis, AEP was diluted 500-fold and 20,000-fold in AES. For ferulic acid, AEP and AES were diluted 100-fold and 1000-fold, respectively.

| No. | Sugar | Ret. Time^a^ (min) |
| --- | --- | --- |
| 1 | Fucose | 3.83 |
| 2 | Rhamnose | 6.33 |
| 3 | Arabinose | 7.33 |
| 4 | Galactose | 9.42 |
| 5 | Glucose | 10.17 |
| 6 | Xylose | 11.25 |
| 7 | Fructose | 13.33 |
| 1 | Mannose | 11.08 |

Supplementary Table Ⅰ. Monosaccharide analysis of standard.

^a^Retention time

Supplementary Table Ⅱ. Monosaccharide analysis of AEP.

| No. | Sugar | Ret. Time^a^ (min) | Amount (mg/mg) |
| --- | --- | --- | --- |
| 1 | Arabinose | 7.42 | 0.001 |
| 2 | Galactose | 9.42 | 0.425 |
| 3 | Glucose | 10.17 | 0.002 |
| 4 | Mannose | 11.08 | 0.304 |

^a^Retention time

Supplementary Table Ⅲ. Monosaccharide analysis of AES.

| No. | Sugar | Ret. Time^a^ (min) | Amount (mg/mg) |
| --- | --- | --- | --- |
| 1 | Rhamnose | 6.25 | 0.004 |
| 2 | Arabinose | 7.42 | 0.002 |
| 3 | Galactose | 9.42 | 0.084 |
| 4 | Glucose | 10.17 | 0.024 |
| 5 | Mannose | 11.08 | 0.001 |

^a^Retention time

Supplementary Table Ⅳ. Results of indicator analysis (GABA, protocatechuic acid, and ferulic acid) of AEP.

| Compound | Amount (mg/kg) | LOQ^a^ (mg/kg) |
| --- | --- | --- |
| GABA | 131.57 | 0.01 |
| Protocatechuic acid | 13.57 | 0.01 |
| Ferulic acid | 0.72 | 0.01 |

^a^Limit of quantitation

| Compound | Amount (mg/kg) | LOQ^a^ (mg/kg) |
| --- | --- | --- |
| GABA | 4576.66 | 0.01 |
| Protocatechuic acid | 532.47 | 0.01 |
| Ferulic acid | 28.39 | 0.01 |

Supplementary Table Ⅴ. Results of indicator analysis (GABA, protocatechuic acid, and ferulic acid) of AES.

^a^Limit of quantitation

**
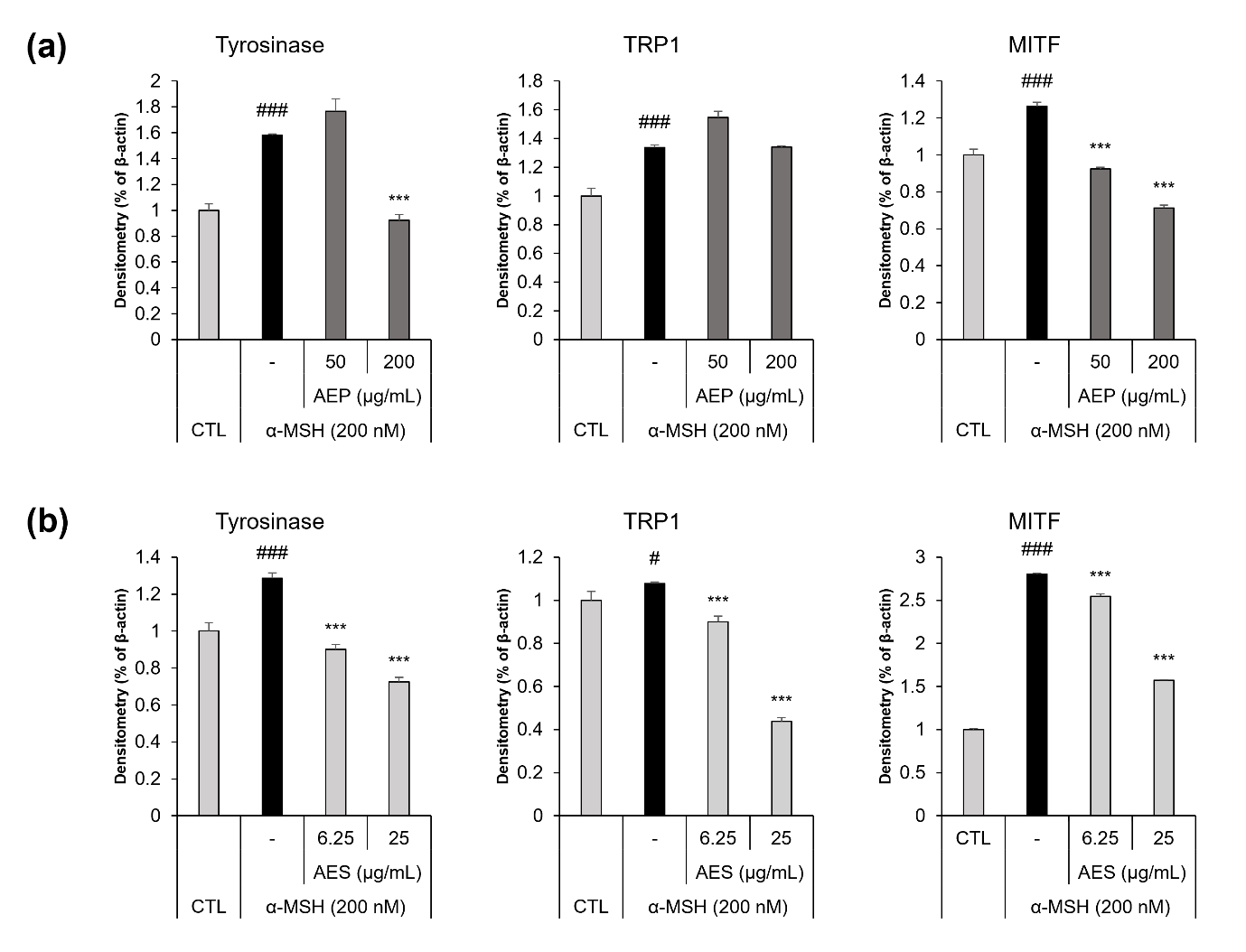
**

**Supplementary Figure 1.** AEP and AES regulate the protein level of melanogenic proteins in α-MSH-stimulated B16F10 cells. (a), (b) Densitometry of Tyrosinase, TRP1, and MITF in AEP (50, 200 μg/mL), AES (6.25, 25 μg/mL) treated α-MSH-stimulated B16F10 cells. (# = p < 0.05, ### = p < 0.001, vs. control group, *** = p < 0.001 vs. α-MSH treatment group)

**
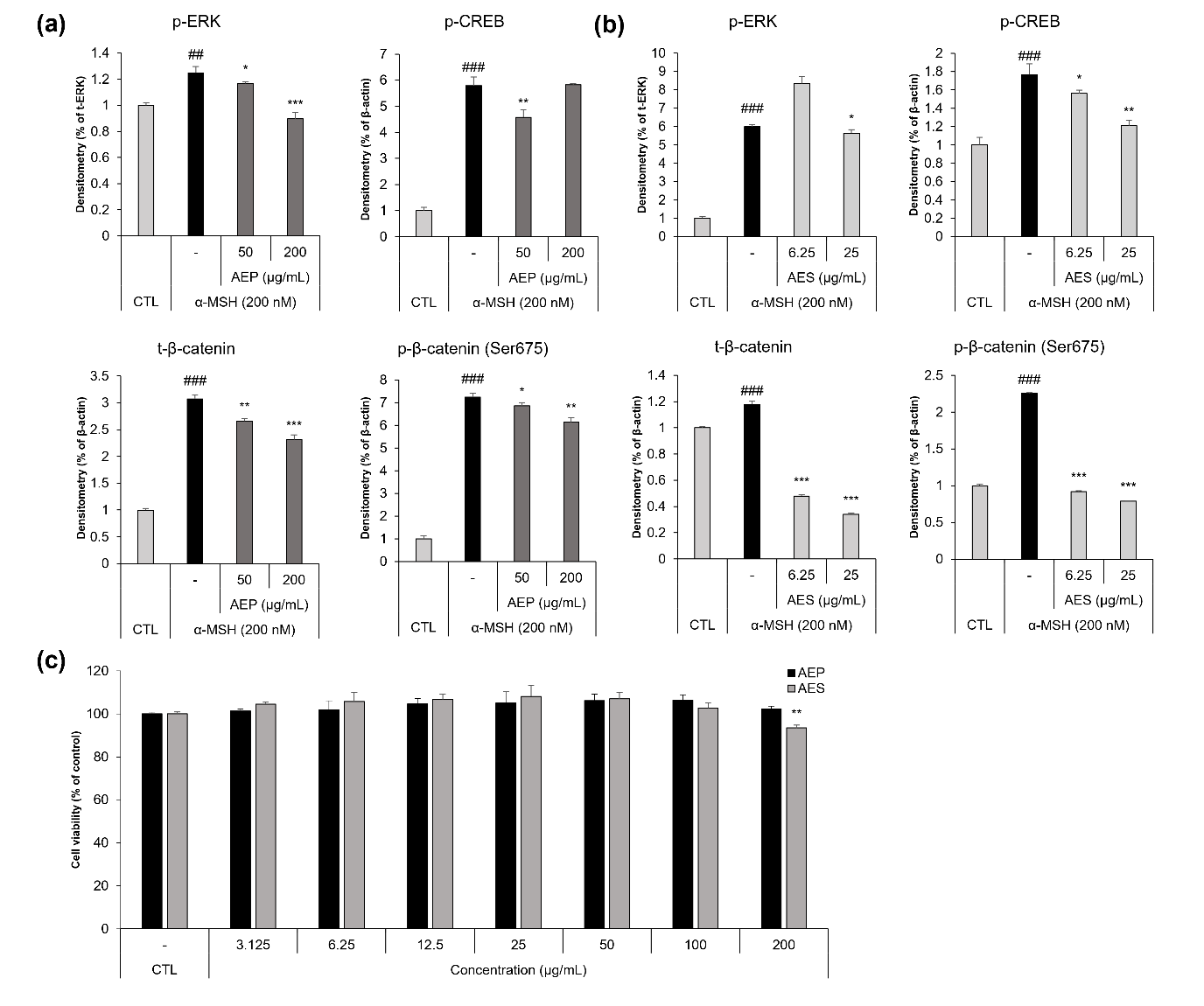
**

**Supplementary Figure 2.** Determination of molecular mechanisms of AEP and AES. (a), (b) Densitometry of p-ERK, p-CREB, t-β-catenin, and p-β-catenin (Ser675) in AEP (50, 200 μg/mL), AES (6.25, 25 μg/mL) treated α-MSH-stimulated B16F10 cells. (c) The cytotoxicity of AEP and AES on MNT-1 cells were determined by using WST-8 assay. Cells were treated with AEP or AES in a concentration range of 3.125-200 μg/mL for 96 h. ((a), (b): ## = p < 0.01, ### = p < 0.001, vs. control group, * = p < 0.05, ** = p < 0.01, *** = p < 0.001 vs. α-MSH treatment group, (c): ** = p < 0.01 vs. control group)
